# XPRS: a tool for interpretable and explainable polygenic risk score

**DOI:** 10.1093/bioinformatics/btaf143

**Published:** 2025-03-31

**Authors:** Na Yeon Kim, Seunggeun Lee

**Affiliations:** Graduate School of Data Science, Seoul National University, 1 Gwanak-ro, Seoul, 08826, South Korea; Graduate School of Data Science, Seoul National University, 1 Gwanak-ro, Seoul, 08826, South Korea

## Abstract

**Summary:**

The polygenic risk score (PRS) is an important method for assessing genetic susceptibility to diseases; however, its clinical utility is limited by a lack of interpretability tools. To address this problem, we introduce eXplainable PRS (XPRS), an interpretation and visualization tool that decomposes PRSs into genes/regions and single nucleotide polymorphism (SNP) contribution scores via Shapley additive explanations (SHAPs), which provide insights into specific genes and SNPs that significantly contribute to the PRS of an individual. This software features a multilevel visualization approach, including Manhattan plots, LocusZoom-like plots, and tables at the population and individual levels, to highlight important genes and SNPs. By implementing with a user-friendly web interface, XPRS allows for straightforward data input and interpretation. By bridging the gap between complex genetic data and actionable clinical insights, XPRS can improve communication between clinicians and patients.

**Availability and implementation:**

The XPRS software is publicly available on GitHub at https://github.com/nayeonkim93/XPRS and can see the demo through our cloud-based web service at https://xprs.leelabsg.org/.

## 1 Introduction

The polygenic risk score (PRS) summarizes the genetic contribution to complex traits by calculating a weighted sum of risk alleles. PRS has emerged as a promising tool for assessing disease susceptibility (Bakshi *et al.* 2023, Deutsch *et al.* 2023, Honigberg *et al.* 2023, Wang *et al.* 2023). However, the explainability of PRS is crucial for its application in clinical settings. Explaining which factors contribute to the predicted risk can increase the reliability of the predictions and help users trust the machine learning system (Ribeiro *et al.* 2016, Lundberg and Lee 2017, Shrikumar *et al.* 2017). In addition, this approach can facilitate communication between key stakeholders, including clinicians and patients.

We introduce eXplainable PRS (XPRS), software that increase the explainability of PRSs by decomposing them into genes/regions and variant contribution scores. Although the PRS model is typically linear, it involves hundreds of thousands or even millions of genetic variants, making it challenging to determine which variants are driving the risk. The functions of most genetic variants remain unknown, making variant-level interpretation particularly difficult. XPRS addresses this by mapping variants to genes or regions, increasing interpretability using Shapley additive explanations (SHAPs) (Lundberg and Lee 2017) to calculate gene contributions.

Visualization is a critical component of explainability. XPRS includes Manhattan plots, and tables for population-level insights, and individual-level visualizations to highlight key genes. LocusZoom-like plots provide variant-level information, offering a detailed view of genetic variants affecting an individual's risk profile.

## 2 Methods

**Figure 1.**
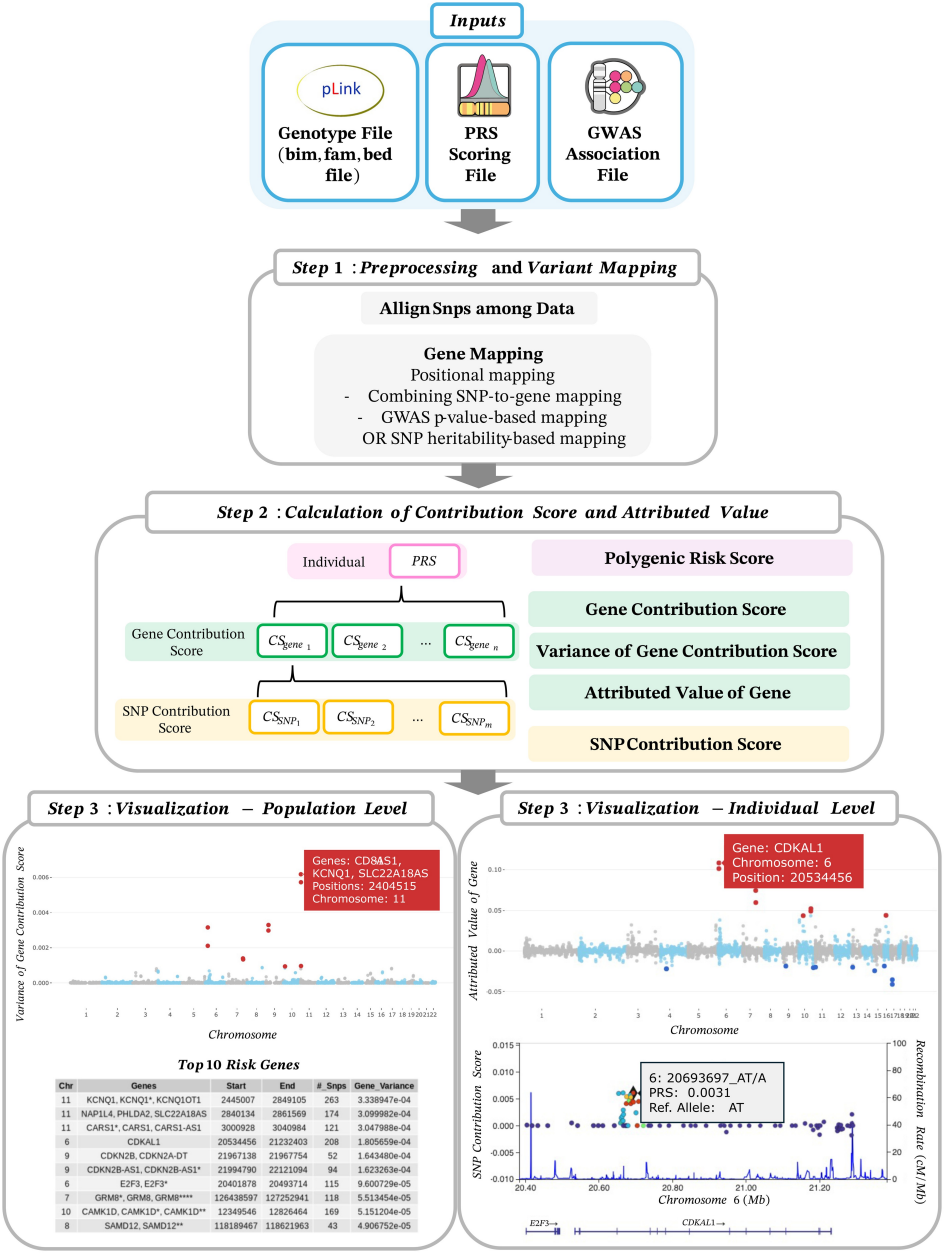
Overview of eXplainable PRS (XPRS). This figure provides an overview of XPRS. Input: Genotype files, PRS scoring files, and GWAS association files. Step 1: Preprocessing and variant mapping: SNPs are aligned and mapped to genes via positional mapping, combined SNP-to-gene mapping, and GWAS *P*-value- or SNP heritability-based mapping. Step 2: Calculation: After mapping, the polygenic risk score (PRS), gene contribution score (CS_gene_), variance of the gene contribution score, attributed value of the gene (A_gene_), and SNP contribution score (CS_SNP_) are computed in this regionizing step. Step 3: Visualization. Population level: Manhattan plot of significant risk genes on the basis of variance in gene contribution scores. Individual level: Density plots, gene-based Manhattan plots, and LocusZoom-like plots of the PRSs of individuals and their specific genetic contributions.

Figure 1 shows an overview of XPRS, which consists of mandatory inputs and three main steps. First, it preprocesses and maps variants to genes using multiple mapping techniques. Next, it calculates contribution scores showing how specific genes or SNPs drive the PRS. Finally, XPRS provides multilevel visualizations for both population‐ and individual‐level interpretation.

### 2.1 Three mandatory inputs

XPRS requires three input files: a genotype file (binary PLINK format), a PRS scoring file, and a GWAS association file. The PRS scoring file contains SNPs, including IDs, effect alleles, and beta coefficients, available from the PGS catalog. To minimize LD-related inflation in effect sizes, we recommend using PRS scoring files generated by LD-aware PRS methods. The GWAS association file is derived from the GWAS Catalog and contains a list of genes mapped to significant variants reported in previous GWAS studies. For accurate PRS estimation, when using a cohort genotype file, all individuals must belong to the same ancestry to prevent biases due to population stratification. If using an individual genotype file instead, the selected reference genotype file must match the individual's ancestry to ensure proper SNP effect size estimation. More details on these requirements are provided in the Supplementary Data.

### 2.2 Three main processes

XPRS consists of three main steps.

**Step1: Preprocessing and variant mapping**

The first step includes aligning SNPs across data sources, standardizing the PRS, and adjusting beta coefficients accordingly and mapping them to corresponding genes. The mapping protocol follows a sequential approach to ensure comprehensive SNP-to-gene assignments. First, positional mapping assigns SNPs to nearby genes based on genomic proximity with a default 200 kb window size, which can be adjusted by the user. Next, combining SNP-to-Gene (cS2G) mapping (Gazal *et al.* 2022) integrates multiple functional genomics datasets such as eQTL analysis and Hi-C interactions to further link SNPs to genes that are not established in the proximity-based approach. At this stage, a single SNP may be assigned to multiple genes due to both the defined window size in positional mapping and functional connections from cS2G. Finally, to ensure that all remaining SNPs are mapped to meaningful loci, we apply GWAS *P*-value or SNP heritability-based mapping. To enhance computational efficiency and highlight significant risk genes, the analysis incorporates a “regionizing” step, consolidating genes with overlapping SNP profiles into distinct regions based on shared SNP content. Further details on the mapping process are provided in the Supplementary Data.

**Step 2: Calculation of the contribution score and attributed value**

The second step involves calculating the gene contribution score ${(CS}_{\mathrm{gene}})$ the attributed value of genes (A_gene_), and the SNP contribution score $({CS}_{\mathrm{SNP}}$), to accurately determine which genes or SNPs contribute to an increased PRS.

*Gene contribution score (CS_gene_) and its variance*: This score quantifies how much each gene contributes to increasing the individual PRS and is calculated by summing the weighted risk alleles mapped to genes:

(1) $${CS}_{{\mathrm{gene}}_{j}}=\;\sum_{i\in {\mathrm{gene}}_{j\;}}{\hat{\beta }}_{\mathrm{std},i}\;\times \;G_{i}^{\prime }\;$$

 

$$\mathrm{where}\;G_{i}^{\prime }=G_{i}-p_{i}$$

where ${\hat{\beta }}_{\mathrm{std},i}$ is the standardized effect size (i.e. weight) of SNP *i*${,\;G}_{i}$ is the individual genotype for SNP *i*, and $p_{i\;}$ is the allele frequency of SNP *i*. Since the adjusted beta coefficients are used, the genotype data are recalculated by subtracting the allele frequency. The gene contribution score reflects how much each gene contributes to the overall PRS, providing a measure of the effect of each gene on disease risk. Additionally, we calculate the variance of ${CS}_{{\mathrm{gene}}_{j}}$ across the population to identify genes that significantly contribute to the PRS among the population. Note that the variance of ${CS}_{{\mathrm{gene}}_{j}}$ is equivalent to the heritability due to gene j.

*Attributed value of genes (A_gene_)*: This value identifies genes that increase disease risk by comparing the contribution score of a gene to the average contribution score in the population:

(2) $${\;A}_{{\mathrm{gene}}_{j}}={CS}_{{\mathrm{gene}}_{j}}-\bar{{CS}_{{\mathrm{gene}}_{j}}}\;$$

where $\bar{{CS}_{{\mathrm{gene}}_{j}}}$ is the mean contribution score of ${CS}_{{\mathrm{gene}}_{j}}$ across all individuals in the population. The attributed value highlights genes with higher-than-average contributions to the PRS, revealing those that significantly elevate disease risk within individuals. We note that $A_{{\mathrm{gene}}_{j}}$ is the SHAP value in a linear model (Lundberg and Lee 2017). For a detailed derivation demonstrating this equivalence, see Supplementary Data.

*SNP contribution score (CS_SNP_)*: This score quantifies the contribution of each SNP to the PRS of an individual. It is calculated by multiplying the standardized effect size (${\hat{\beta }}_{\mathrm{std},i}$) and the number of risk alleles ($G_{i}^{\prime }$) that an individual carries for an SNP *i*:

(3) $${CS}_{{\mathrm{SNP}}_{i}}=\;{\hat{\beta }}_{\mathrm{std},i}\;G_{i}^{\prime }\;$$

These calculations provide a detailed understanding of the PRS model. The gene contribution score quantifies the overall impact of each gene on the PRS, whereas the attributed value distinguishes genes with significant contributions compared with the population average, and the SNP contribution score breaks down the gene impact into specific SNP effects.

**Step 3: Visualization**

The final step involves visualization to elucidate disease susceptibility at both the individual level and population level. The derived metrics from the PRS calculations are presented through a multilevel visualization approach. At the population level, Manhattan plots and tables are used to identify important genes on the basis of the highest variance of ${CS}_{\mathrm{gene}}$, which highlights which genes or regions drive the PRS model. Instead of applying a fixed threshold, the genes are ranked from highest to lowest variance of ${CS}_{\mathrm{gene}}$, and the top 10 genes with the highest variance are highlighted in red in the Manhattan plot. At the individual level, visualization includes Manhattan and LocusZoom-like plots, which show which gene/regions and SNPs drive high or low values of the PRS score of an individual. Gene contribution scores are ranked from highest to lowest, with the top 10 genes with the highest contribution highlighted in red and the bottom 10 genes with the lowest contribution highlighted in blue in the Manhattan plot.

## 3 Results

### 3.1 Visualizing the contribution of genes or regions to the PRS in the population

To identify which genes contribute the most to the PRS in a population, we visualized the variance in the gene contribution scores. The input data for this analysis included genotype files from the 1000 Genomes Project (Auton *et al.* 2015), with 503 samples and 80 855 722 variants from the East Asian population. The PRS scoring file was obtained from the study by Kim *et al.* (2024), which evaluated the PRS for type 2 diabetes mellitus in the Korean population. Additionally, the GWAS association file was obtained from the GWAS Catalog (https://www.ebi.ac.uk/gwas/) for type 2 diabetes.

Supplementary Figure S2 presents a Manhattan plot and a table based on the analysis. The Manhattan plot highlights the importance of risk genes associated with type 2 diabetes, where each point represents a gene and its variance in gene contribution score—higher variance indicates greater importance as a risk factor. The accompanying table lists significant genes, the number of mapped SNPs per gene, and their respective variances. KCNQ1 and KCNQ1OT1 are well-established risk genes for type 2 diabetes (Travers *et al.* 2013), and in our analysis, they exhibit the greatest variances in gene contribution score, underscoring their significant role in susceptibility to type 2 diabetes.

### 3.2 Visualizing the impact of genes and SNPs in an individual

To trust the PRS results and effectively communicate with patients, it is crucial to pinpoint which specific genes and SNPs contribute to the PRS of an individual. By visualizing the attributed values of gene and SNP contribution scores for each individual, we to increase the explainability of the PRS score. The example individual, HG00464, is obtained from the 1000 Genomes Project. Supplementary Figure S3 shows the individual PRS within the population via a density plot, which shows the position of an individual relative to high or low genetic risk for type 2 diabetes. This individual has a high PRS, which is in the top 2% of the population.

The figure includes a Manhattan plot and two tables to highlight specific genes that elevate or reduce the PRS. For example, the CDKAL1 gene, a well-known T2D-associated gene (Steinthorsdottir *et al.* 2007), had the highest attributed value. If this individual had the population average contribution value, the PRS score would be lower by 0.028. Additionally, the LocusZoom-like plot displays the detailed visualization of SNP contributions within the gene. For example, individual HG00464 carries 208 alleles in CDKAL1, which increases its PRS value. These figures enable the explanation of the PRS of an individual at both the gene level and SNP level, facilitating effective communication about their genetic risk.

On the other hand, in Supplementary Fig. S4, the example individual, HG01816, has a significantly lower PRS than the population average does, indicating a lower genetic predisposition to type 2 diabetes. This figure also includes a Manhattan plot and table to emphasize specific genes that contribute minimally or even reduce the PRS. For example, KCNQ1, known as a diabetes-related gene (Erfani *et al.* 2020), had the lowest attributed value. Additionally, the KCNQ1-AS1 gene, another gene associated with type 2 diabetes, had the second lowest attributed value, further contributing to the reduced PRS of the individual.

## 4 Conclusion

The XPRS tool enhances the interpretability of PRS by providing detailed contribution scores for individual genes and SNPs. By breaking down the PRS into its granular components, XPRS improves the explainability of these scores. Notably, identifying which factors contribute to genetic risk increases the reliability of predictions and strengthens user trust in the system. Furthermore, this approach will aid in effectively communicating genetic risk factors to key stakeholders, including clinicians and patients, ensuring that complex genetic information is more accessible and actionable in a clinical setting.

## Supplementary Material

btaf143_Supplementary_Data

## Data Availability

The data underlying this study are available in the article’s Supplementary data.
