# Supplementary material for: XPRS: a tool for interpretable and explainable polygenic risk score
Source: Bioinformatics. 2025 Mar 31;41(4):btaf143. doi: 10.1093/bioinformatics/btaf143 (PMC12043004; doi:10.1093/bioinformatics/btaf143)
Supplement: btaf143_Supplementary_Data [file btaf143_supplementary_data.docx]

**Supplementary Data**

**for**

**XPRS:** **A Tool for Interpretable and**

**Explainable Polygenic Risk Score**

Na Yeon Kim^1^, Seunggeun Lee^1,*^

^1^ Graduate School of Data Science, Seoul National University, Seoul, South Korea

**Corresponding author**

Seunggeun Lee
Graduate School of Data Science
Seoul National University, Seoul, South Korea
[lee7801@snu.ac.kr](mailto:lee7801@snu.ac.kr)

**Methods**

**Inputs**

Our software, XPRS, requires the upload of three mandatory input files: a genotype file, a PRS scoring file, and a GWAS association file. The genotype file must be in binary PLINK format. The PRS scoring file, is available for download from the PGS catalog (<https://www.pgscatalog.org/>) or can be generated via GWAS summary statistics and reference files with various PRS construction methods. To minimize LD-related inflation in effect sizes, users are recommend to use PRS scoring files generated by LD-aware PRS methods. Moreoever, if users generate the PRS scoring file via PRS construction methods, they can optionally use GWAS summary statistics to incorporate GWAS p-values for more accurate mapping information.

The GWAS association file contains a curated list of genes mapped to significant variants reported in previous GWAS studies, rather than raw summary statistics, which can be downloaded from the GWAS Catalog (<https://www.ebi.ac.uk/gwas/>). It is important to note that the GWAS association file is distinct from GWAS summary statistics, as the two are sometimes confused due to their similar terminology. In the population-level visualization, using the GWAS association file, XPRS ensures that only genes mapped to significant variants are displayed, rather than all genes within a region, providing a clearer representation of risk genes. The concept of 'regions' will be elaborated in subsequent methodological steps.

**Parameters**

XPRS allows the modification of three key parameters to optimize computational efficiency and accuracy:

- CPU nodes: Users can adjust the number of CPU nodes, with a default setting of 8. When SNPs are mapped to genes, we use the parallel package in R for parallel computing to achieve faster computation.
- Top SNP heritability percentage: Users can specify the percentage of SNPs with the highest heritability to be included in the analysis. SNP heritability is calculated using the equation $heritability= \beta^{2}\times MAF\times(1-MAF)$. This selection is crucial not only for computational efficiency but also to ensure that only the most informative SNPs contribute to gene scores, effectively reducing noise and limiting the influence of redundant SNPs within LD blocks. By default, up to 100,000 SNPs are included. If users wish to include fewer, they can specify a percentage indicating how much of the top SNPs should be included based on heritability. However, the total number of included SNPs will not exceed 100,000.
- Window size: The default genomic window size is 200 kb, which is used when SNPs are mapped to genes through positional mapping on the basis of an annotation file. Additionally, this window size is also applied in GWAS p-value or SNP heritability-based mapping to ensure consistent SNP-to-gene assignments across different mapping strategies.

These parameters increase the flexibility and performance of XPRS, allowing for efficient computation and improved interpretation of risk genes and SNPs.

**Three main processes**

**Step 1: Preprocessing and variant mapping**

Initial preprocessing involves SNP alignment between the genotype file and the PRS scoring file to ensure the correct inclusion of variants in the PRS. This process includes making all beta values positive and correspondingly switching the A1 and A2 allele positions to ensure that the effect sizes are consistently oriented. Additionally, SNP heritability-based filtering is applied at this stage to retain only the most informative SNPs, removing redundant SNPs with lower heritability that may introduce noise. This ensures that only SNPs with strong heritability signals contribute to the PRS calculation. The raw PRS is calculated as a weighted sum of risk alleles via positive beta coefficients. The PRS is then standardized to achieve a mean of zero and a standard deviation of one. The beta coefficients are subsequently adjusted on the basis of the standardized PRS to ensure that the contribution scores derived in subsequent steps accurately reflect the standardized risk contributions. This step is followed by a three-phase gene mapping protocol:

1. **Positional mapping**: We mapped SNPs to genes on the basis of their genomic position via refGene annotation (<https://hgdownload.soe.ucsc.edu/goldenPath/hg38/database/>) with a default 200kb window size (user adjustable). In cases where a gene exhibits multiple start and end positions owing to alternative splicing or isoforms, distinct start‒end coordinates are denoted with asterisks (e.g., Gene*, Gene**, and Gene***). In Supplementary Fig 1, SNPs in red boxes represent positional mapping, where a SNP may be mapped to multiple genes within the predefined window size.

2. **Combining SNP-to-Gene (cS2G) mapping**(Gazal, et al., 2022): This employs several linkage methodologies, including expression quantitative trait loci (eQTL) analysis, enhancer‒gene interactions, and promoter capture Hi-C (PCHI-C) techniques, to map SNPs to genes. The cS2G file used for this step was downloaded from <https://zenodo.org/records/6354007>. SNPs in blue boxes illustrate cS2G mapping, where SNPs can be assigned to multiple genes (Supplementary Fig 1).

3. **GWAS p-value or SNP heritability-based mapping**: For SNPs not mapped in the previous two steps, we use GWAS p-value significance or SNP heritability estimates. When GWAS summary statistics are provided, we identify the index SNP with the lowest p-value first, assigning neighboring SNPs to the index SNP to form a region within a predefined genomic window with a default 200kb window size, which can be adjusted by the user. This process continues until all SNPs are mapped. If GWAS summary statistics are not available, we calculate SNP heritability via the equation $heritability= \beta^{2}\times MAF\times(1-MAF)$, where β is the effect size from the PRS model and MAF is the minor allele frequency. The SNPs with the highest heritability are mapped first, following the same regional assignment process until all the SNPs are mapped. The green box, in the Supplementary Fig 1, highlights GWAS p-value or SNP heritability-based mapping, where the index SNP within a predefined genomic window is used to iteratively map neighboring SNPs.

Following the mapping protocol, the analysis incorporates a 'regionizing' step which is the process consolidates genes with overlapping SNP profiles into distinct regions on the basis of shared SNP content. For example, genes with identical SNP compositions —i.e., those with an identical SNP composition— are combined and calculated as single entities. In cases in which genes share a significant proportion of SNPs (e.g., two-thirds overlap), they are grouped into the same region. Within each region, we identified the gene with the highest variance in the gene contribution score to represent the risk gene. For example, consider Region B in the Supplementary Fig 1, which contains Gene 2, Gene 3, and Gene 4 due to their substantial SNP overlap. Suppose the variance of the gene contribution scores for these genes is as follows:

- $Var\left( {CS}_{{Gene}_{2}} \right)=$ 3
- $Var\left( {CS}_{{Gene}_{3}} \right)=$ 1
- $Var\left( {CS}_{{Gene}_{4}} \right)=$ 5

In such cases, we report only Gene 4 in the results, as it exhibits the highest variance in gene contribution score ($Var\left( {CS}_{gene} \right)$). This approach ensures that we highlight the most influential gene within a region, improving interpretability and reducing redundancy.

**Step 2: Calculation of the contribution score and attributed value**

To clarify how our gene-level contribution score and attributed value align with the SHAP framework in a linear model(Lundberg and Lee, 2017), we provide the following derivation. The equations below detail how we arrive at this result, beginning with the general form of a linear model and concluding with our gene-level formulation.

$$f\left( x \right)= \sum_{j=1}^{M} w_{j}x_{j}+b$$

the SHAP value for feaure *j* can be expressed as

$$\phi_{j}\left( f, x \right)= w_{j}(x_{j}-E[x_{j}])$$

Essentially, each feature’s contribution depends on how much its current value $x_{j}$ deviates from its baseline $E[x_{j}]$, weighted by the corresponding coefficient $w_{j}$. In our formulation, each gene *j* has a contribution score defined by:

$${CS}_{{gene}_{j}}= \sum_{i \in{gene}_{j}} \hat{\beta}_{std, i} \times G_{i}^{'}$$

where $\hat{\beta}_{std, i}$​ is the standardized effect size (analyogous to the coefficient $w_{j}$in the linear model), and $G_{i}^{'}$is the standardized genotype value (analogour to $x_{j}$). We then measure the gene’s attributed value by substracting its baseline contribution.

$$A_{{gene}_{j}}={CS}_{{gene}_{j}}-\bar{{CS}_{{gene}_{j}}}$$

This differences captures how much gene *j*’s current contribution deviated from its expected level, mirroing the linear SHAP notion of $w_{j}(x_{j}-E[x_{j}])$.

**Implementation**

The XPRS software is designed to be both user friendly and efficient. We developed a web interface using Flask, enabling users to input data easily through a web page. The gene contribution scores are calculated using C++ for optimal performance, whereas R is employed for data preprocessing and visualization. This integrated approach ensures accessible and efficient handling of complex computational tasks.

**Execution**

The XPRS platform was developed via a web interface with Flask, a lightweight WSGI web application framework in Python. To start the XPRS platform, the following environment variables need to be set:

export FLASK_APP=app

export FLASK_ENV=development

export FLASK_DEBUG=1

flask run -p 5000

After executing these commands, a web browser will automatically open, launching the XPRS interface, allowing users to interact with the platform easily, similar to any standard web application.

**Web interface**

XPRS comprises four main sections accessible from the left panel: Home, Tutorial, Run, and Links & Data. The "Home" section provides a brief introduction to XPRS. The "Tutorial" section offers an overview of the software and the input format to ensure that users can easily understand the required data formats. The "Links & Data" section provides useful external links for downloading required input files such as those from the PGS Catalog and the GWAS catalog.

In the "Run" section, there are four tabs for different genotype data cases. Case 1 considers a scenario in which users have a large cohort genotype file, which can be used for both population reference genotypes and genotypes of each individual for PRS prediction. With the cohort genotype file, XPRS highlights risk genes in the population. Users can conduct individual-level analysis by entering the individual ID (iid). The population-level results are saved as ‘data.rds’, enabling users to rerun analyses via previously processed data by selecting Case 1-1 and uploading ‘data.rds’, which reduces the time required for analysis. To ensure consistency in PRS estimation, users must provide a single-ancestry cohort genotype file. Mixing multiple ancestries within a cohort may lead to biased results due to differences in linkage disequilibrium structure, allele frequencies, and genetic architecture across populations.

For users without a cohort genotype file, Case 2 enables PRS calculation using external reference datasets, such as the 1000 Genomes Project. In this scenario, users upload their individual genotype data to obtain personalized PRS results. To ensure accuracy, the reference dataset must be ancestry-matched to the individual's genetic data. For example, if an individual's genetic data originates from an East Asian population, the reference genotype file should also be from an East Asian reference panel to minimize bias in PRS interpretation. Like Case 1-1, Case 2-1 allows users to upload a previously generated ‘data.rds’ file to efficiently rerun analyses.

Before exiting the web interface, users should rename and save the data.rds file separately if they wish to retain it for future use. Exiting the interface using Ctrl+C will delete the default XPRS-main/output/data.RDS and XPRS-main/data/new_test file, making it unrecoverable. The users should rename both:

- XPRS-main/output/data.RDS (to preserve the processed results)
- XPRS-main/data/new_test (PLINK file)

Renaming these files before exiting ensures that they can be reused in future analyses without loss of data.

**Computational resources**

When the XPRS platform runs on an AMD EPYC 7542 CPU with 8 cores, it takes approximately 5.72 minutes to visualize the contribution of genes to the PRSs for 503 samples with 80,855,722 variants in a population-level analysis. The visualization of the impact of genes and SNPs for an individual is completed in approximately 2.54 seconds.

**Discussion**

Although XPRS incorporates LD-aware PRS beta values and applies a heritability-based SNP filtering step to mitigate linkage disequilibrium (LD) effects, residual LD-driven redundancy may still persist. In particular, correlated SNPs that pass the filtering threshold could contribute overlapping signals to a gene’s score. While the regionizing step further reduces this effect by collapsing overlapping SNP-gene assignments into a single region, XPRS does not explicitly model remaining LD among SNPs beyond these measures. Therefore, users should remain aware that in highly correlated genomic regions, some degree of LD-related inflation could still influence gene-level results, albeit to a lesser extent than methods lacking these safeguards.

**Code availability**

The XPRS software is publicly available on GitHub at <https://github.com/nayeonkim93/XPRS> and can be accessed through our cloud-based web service at <https://xprs.leelabsg.org/>. XPRS integrates LocusZoom for visualization purposes and utilizes PLINK for genome-wide association analyses. LocusZoom is available at <https://github.com/statgen/locuszoom> and PLINK can be accessed at <https://www.cog-genomics.org/plink/1.9/>.

**Data availability**

SNPs are mapped to genes based on their genomic positions using the RefGene annotation from the UCSC Genome Browser (<https://hgdownload.soe.ucsc.edu/goldenPath/hg38/database/>). The cS2G file utilized in this process was obtained from Zenodo (<https://zenodo.org/records/6354007>).

**References**

Gazal, S.*, et al.* Combining SNP-to-gene linking strategies to identify disease genes and assess disease omnigenicity. *Nature Genetics* 2022;54(6):827-836.

Lundberg, S.M. and Lee, S.I. A Unified Approach to Interpreting Model Predictions. *Adv Neur In* 2017;30.

**Suppelemetary Figures**

**
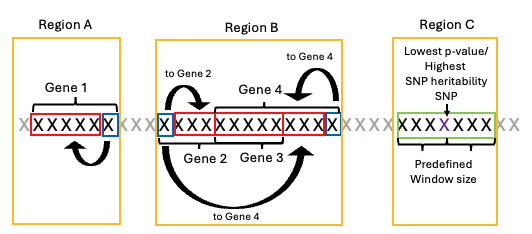
Supplementary Fig 1.** Overview of the three steps SNP-to Gene mapping and regionizing in XPRS. Each **"X"** represents an SNP, and **grey SNPs** indicate those that have been filtered out based on the Top SNP heritability percentage threshold, ensuring that only the most informative SNPs contribute to gene scores. **Red boxes** indicate SNPs mapped to genes using positional mapping. **Blue boxes** highlight combining SNP-to-Gene (cS2G) mapping. **Green boxes** represent GWAS p-value or SNP heritability-based mapping. Following Gene mapping procedure, SNPs are grouped into distinct regions **(highlighted in yellow box)** based on shared SNP content (e.g., two-thirds overlap).

**Supplementary Fig 2.** Contribution of genes to Type 2 Diabetes PRS in the Asian population from the 1000 Genomes Project. **Manhattan plot**: Variance in gene contribution scores across chromosomes. Higher variance indicates greater significance as a risk factor, with notable genes highlighted. **Table**: Significant genes, including chromosomes (chr), gene names, start and end positions, number of SNPs included in mapped genes, and variance in gene contribution scores.

**
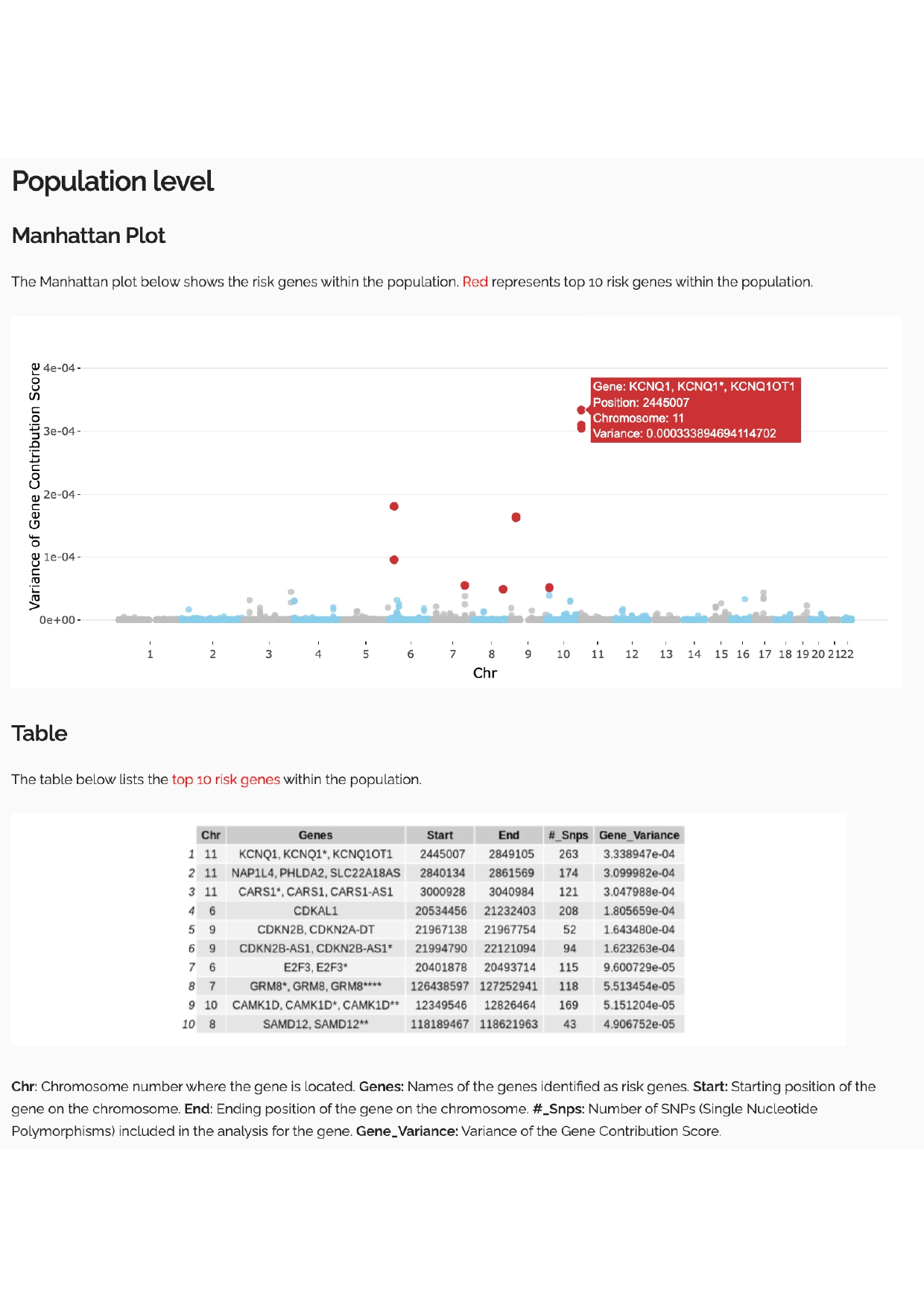
**

**Supplementary Fig 3.** Effect of gene and SNP on an individual PRS from the 1000 Genome Project for type 2 diabetes. High PRS sample **IID**: HG00464. **Density plot**: PRS of the individual within the population distribution, indicating their genetic risk position. **Table: Top 10 risk genes and bottom 10 nonrisk genes within the individual.** **Manhattan plot**: Significant genes contributing to the PRS of the individual, with higher points representing greater impact. **LocusZoom-like plot**: Detailed SNP contributions for the top 10 risk genes and bottom 10 nonrisk genes

**
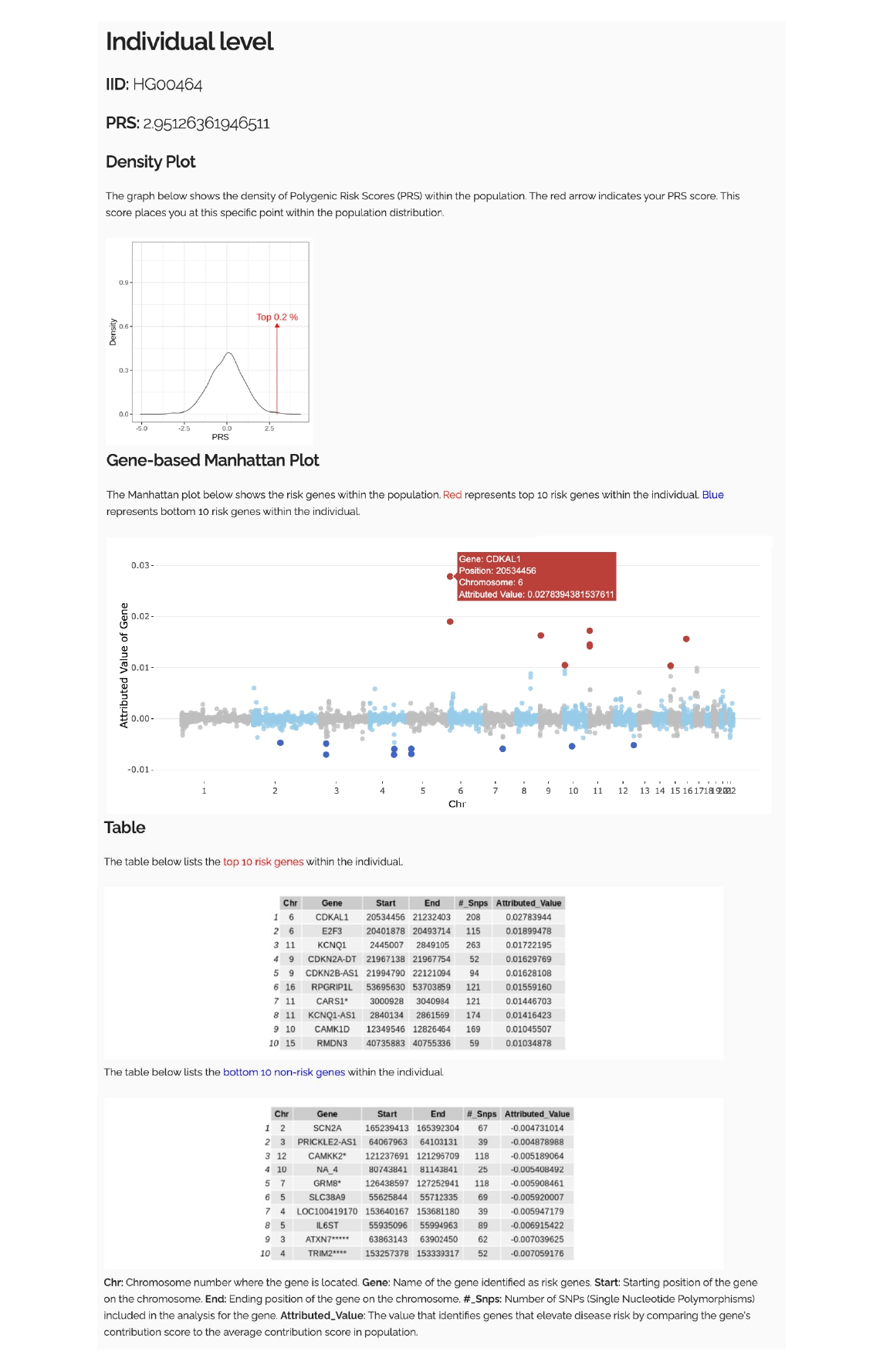
**

**
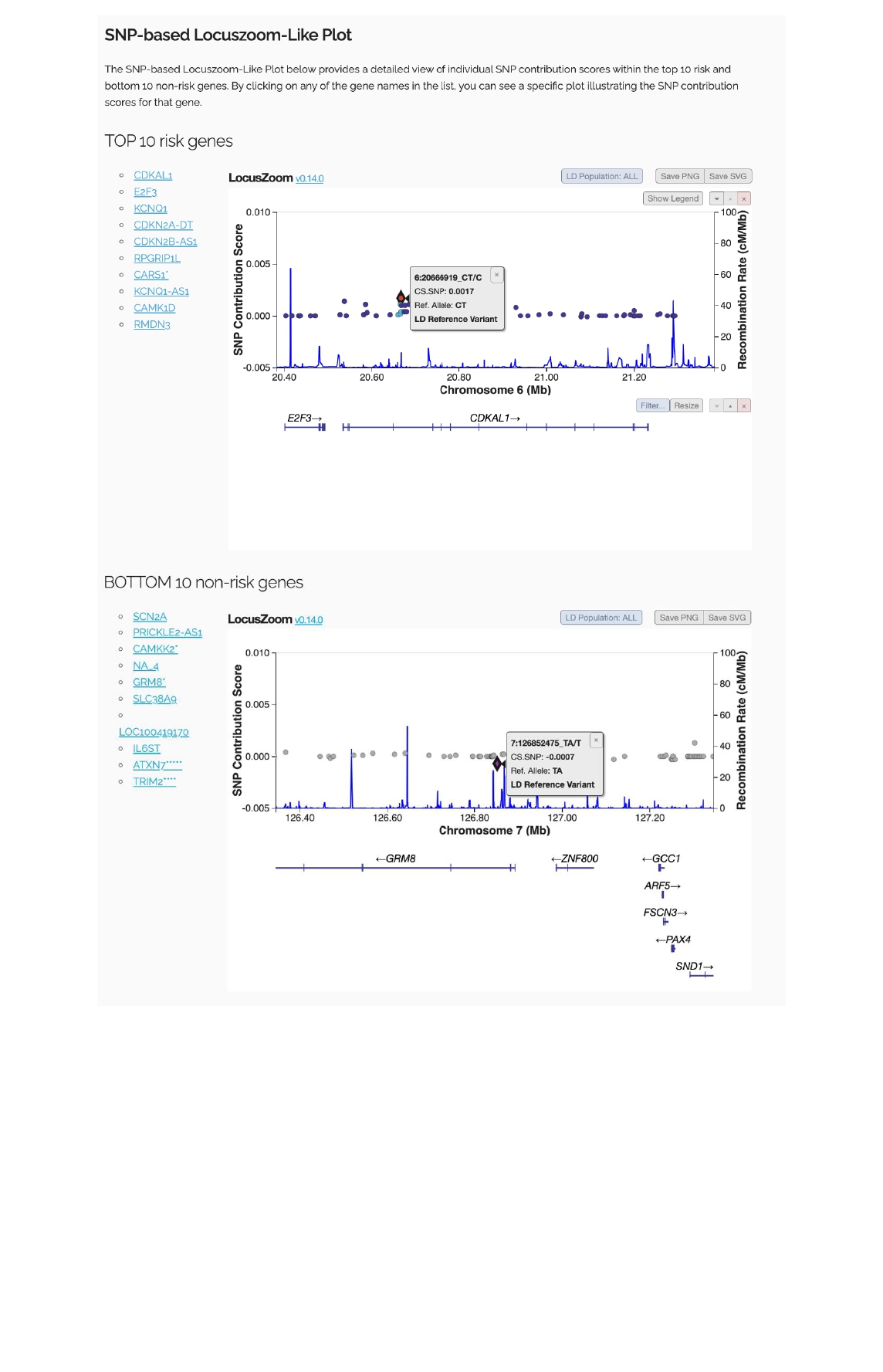
**

**Supplementary Fig 4.** Effect of gene and SNP in an individual PRS from the 1000 Genome Project for type 2 diabetes. Low PRS sample **IID**: HG01816. **Density plot**: PRS of the individual within the population distribution, indicating their genetic risk position. **Table: Top 10 risk genes and bottom 10 nonrisk genes within the individual.** **Manhattan plot**: Significant genes contributing to the PRS of the individual, with higher points representing greater impact. **LocusZoom-like plot**: Detailed SNP contributions for the top 10 risk genes and bottom 10 nonrisk genes

**
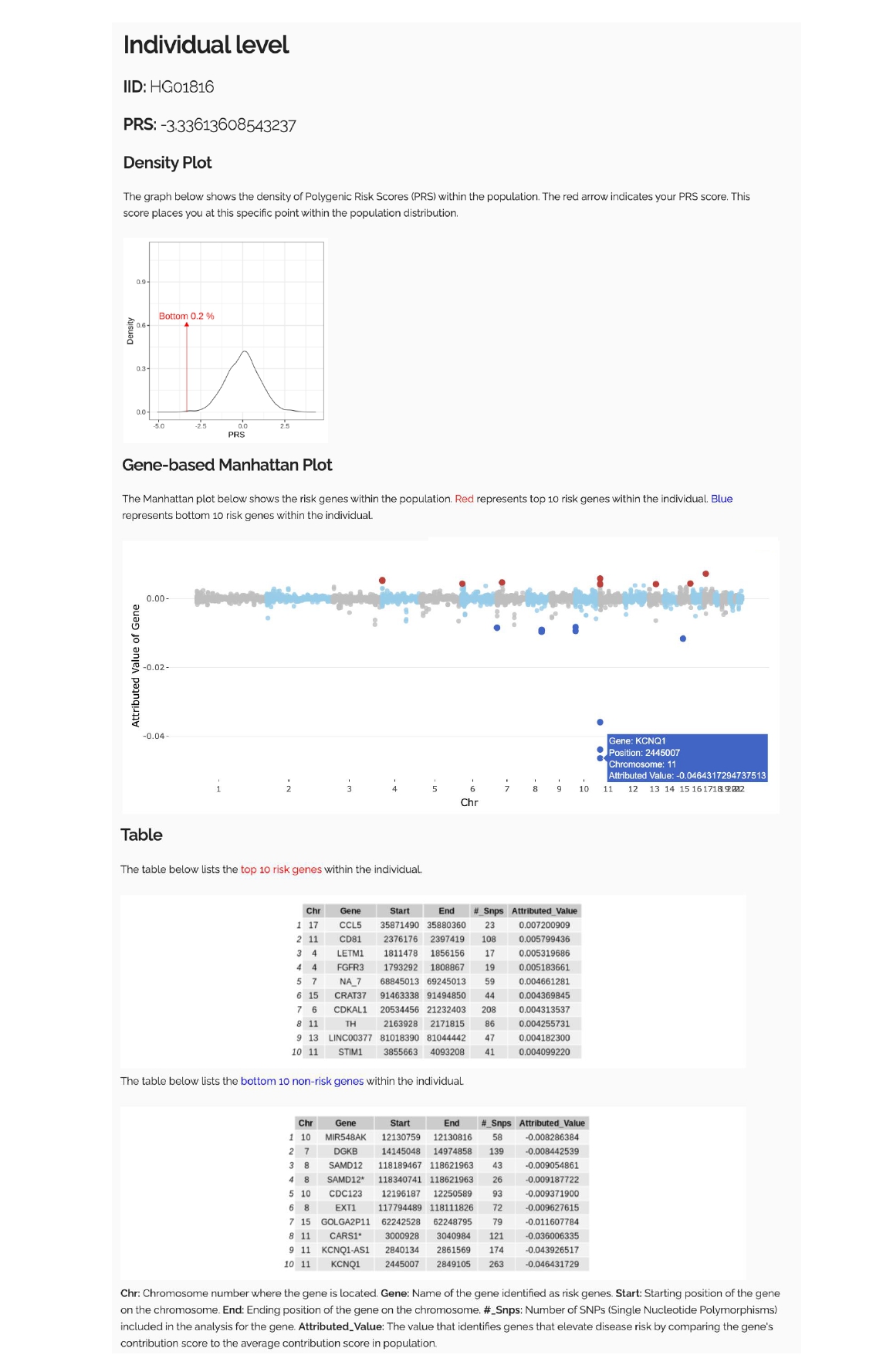
**

**
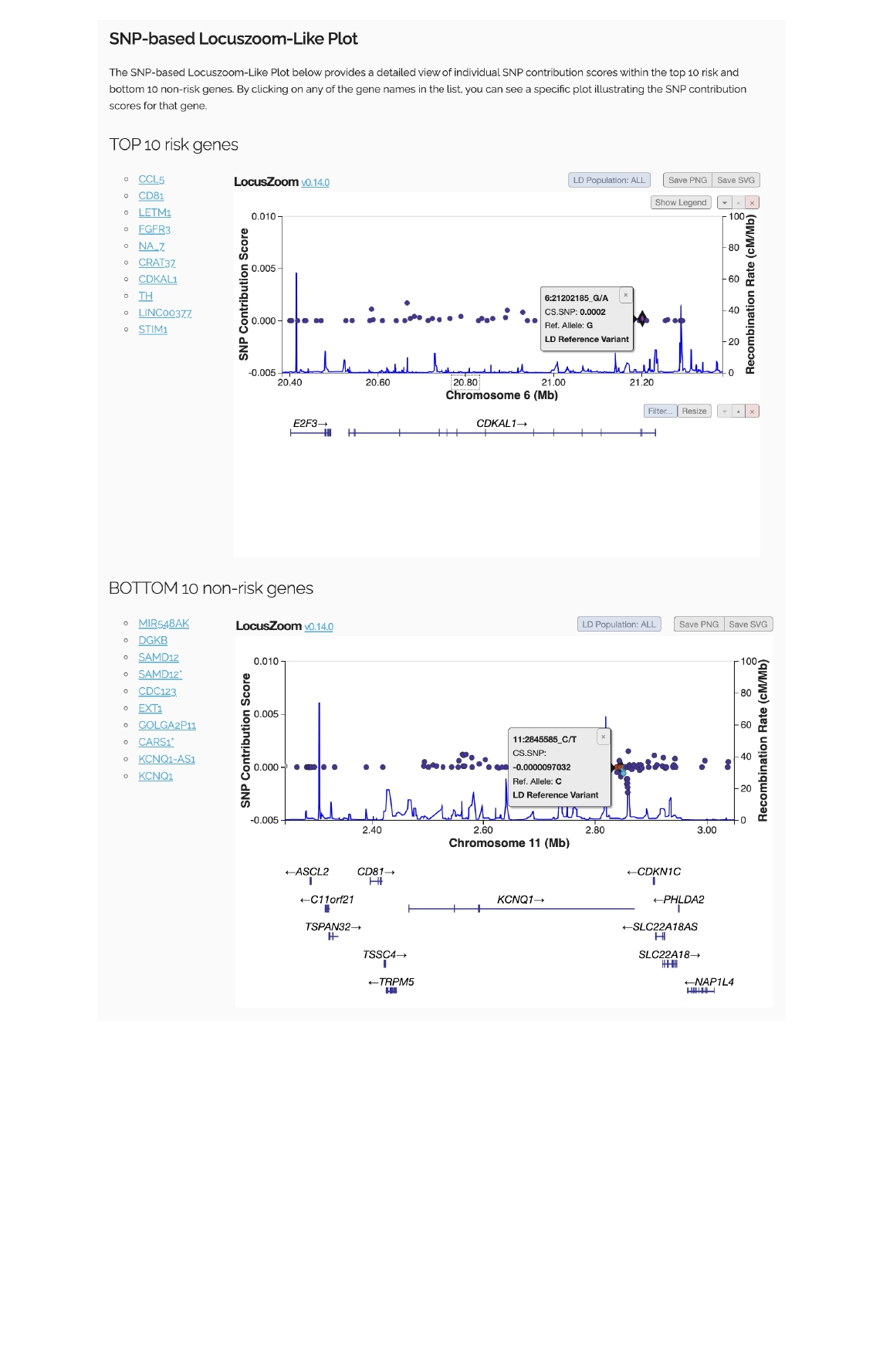
**
